# Experience with selexipag in triple therapy for pulmonary arterial hypertension in Chinese children

**DOI:** 10.1186/s12887-026-06954-9

**Published:** 2026-05-08

**Authors:** Meng Li, Yingchun Wang, Xiaoyu Hu, Haizhao Zhao, Weida Lu, Yuan Ji, Xiaopei Cui

**Affiliations:** 1 Department of Pediatrics, Qilu Hospital of Shandong University, No.107 West Wenhua Road, Jinan, Shandong Province 250012 China; 2 Department of Geriatric Medicine & Laboratory of Gerontology and Anti-Aging Research, Qilu Hospital, Cheeloo College of Medicine, Shandong University, No.107 West Wenhua Road, Jinan, Shandong Province 250012 China

**Keywords:** Pulmonary Arterial Hypertension, Prostacyclin receptor agonist, Paediatric, Selexipag, Adverse reaction, Treatment

## Abstract

**Background:**

Selexipag is an orally effective prostacyclin receptor agonist that has been approved for treating pulmonary arterial hypertension (PAH) in adults but is still used off-label in children. This study aimed to evaluate the efficacy and safety of selexipag as part of triple combination therapy (TCT) with endothelial receptor antagonists (ERAs) and phosphodiesterase-5 inhibitors (PDE5is) in Chinese children with PAH.

**Methods:**

We conducted a retrospective single-centre study including pediatric patients with Group 1 PAH who received selexipag-based TCT at Qilu Hospital of Shandong University from November 2018 to September 2023. A total of 10 pediatric patients were enrolled, with ages ranging from 8.9 to 17.2 years. Clinical data, biomarker levels, and echocardiograms were collected every 6 months.

**Results:**

In total, 10 children (7 females) were enrolled, with a median age of 14.5 years. The median follow-up duration was 29.3 months. During follow-up, 4 patients (40%) died. At the 6-month follow-up, improvements were observed in NT-proBNP levels (*n* = 9), 6-min walk distance (6MWD; *n* = 8) and WHO functional class (WHO-FC; *n* = 4). Among surviving patients, paired analysis revealed that 83.3% (5/6) showed an improvement in WHO-FC (*P* < 0.05), NT-proBNP levels were significantly reduced (*P* < 0.05), and 6MWD exhibited a non-significant increasing trend. No statistically significant changes were observed in echocardiographic parameters. The 1-, 2-, and 3-year transplant-free survival rates were 80%, 70%, and 60%, respectively. Selexipag was generally well tolerated, and no patients discontinued treatment due to adverse events.

**Conclusion:**

In this small retrospective cohort, selexipag-based TCT was associated with improvements in WHO-FC and NT-proBNP levels and acceptable safety in Chinese children with PAH. Nevertheless, our findings are limited by the small sample size and retrospective single-centre design, highlighting the need for larger prospective studies.

**Trial registration:**

Not applicable.

**Supplementary Information:**

The online version contains supplementary material available at 10.1186/s12887-026-06954-9.

## Background

Paediatric pulmonary arterial hypertension (PAH) is a rare and progressive disorder characterized by pulmonary vascular remodelling and sustained elevation of pulmonary vascular resistance, leading to right heart failure and premature mortality [1, 2]. Although advances in targeted therapies have substantially improved survival in children over the past two decades, treatment options remain limited [3–6].

Current pharmacological strategies for PAH primarily target the nitric oxide, endothelin, and prostacyclin signalling pathways [6, 7]. Oral phosphodiesterase-5 inhibitors and endothelin receptor antagonists are approved for paediatric use and constitute the backbone of therapy in many children [8]. In contrast, prostacyclin pathway activation in paediatric PAH has historically relied on parenteral prostacyclin analogues, which, despite proven efficacy, are associated with significant treatment burden and catheter-related complications, limiting their long-term acceptability in children [9, 10].

Selexipag is an orally administered, selective prostacyclin IP receptor agonist that has demonstrated clinical benefit in adult PAH and is approved for adult use in multiple regions [11–13]. In children, however, its application remains off-label [8]. Paediatric PAH requires age-specific evidence due to heterogeneous aetiologies, developmental factors, and age-dependent pharmacokinetics. Emerging evidence from initial reports to recent prospective explorations indicates that selexipag is well-tolerated in children and may improve hemodynamic parameters as part of combination therapy, with pharmacokinetic analyses supporting weight-based dosing [14–20].

While data in Asian children are emerging, including a phase 2 trial in 6 Japanese patients, the experience in Chinese paediatric patients has not yet been reported [21]. This study therefore aimed to retrospectively assess the efficacy and safety of selexipag within triple therapy in Chinese children with PAH.

## Methods

### Study design and patient enrolment

This was a single-centre retrospective study on the experience of the Pulmonary Hypertension (PH) Treatment Centre at the Qilu Hospital of Shandong University in northern China. The primary objective of this study was to retrospectively evaluate the efficacy and safety of selexipag when used as part of triple combination therapy in Chinese children with PAH.

All patients included in our study met the following inclusion criteria: ➀ Pediatric patients aged 2 to 18 years at the time of enrollment; ② Diagnosed with Group 1 PH confirmed by right heart catheterization (RHC); ③ Received selexipag as part of triple combination therapy at our centre between 1 November 2018 and 30 September 2023; ④ Patients were required to have WHO functional class (WHO-FC) II, III or Ⅳ; ⑤ Complete clinical data (including demographic information, treatment records, and efficacy/safety follow-up data) were available. Children with PAH not diagnosed by RHC, or those with severe organic diseases (e.g., severe hepatic or renal dysfunction, malignant tumors) that might influence study outcomes, were excluded. Pre-capillary PH was diagnosed according to the 7th World Symposium on Pulmonary Hypertension (WSPH) in 2024: in children > 3 months of age at sea level, mean pulmonary artery pressure (mPAP) > 20 mmHg, pulmonary artery wedge pressure (PAWP) or left ventricular end-diastolic pressure (LVEDP) ≤ 15 mmHg, and pulmonary vascular resistance index (PVRI) ≥ 3 WU·m^2^ [22].

The study included patients who initiated triple combination therapy (TCT) with selexipag, endothelial receptor antagonists (ERAs), and phosphodiesterase-5 inhibitors (PDE5is) between 1 November 2018 and 30 September 2023 (enrollment period). Upfront TCT (uTCT) was defined as the initiation of triple therapy, including selexipag, ERAs, and PDE5is within 6 weeks of diagnosis. Sequential TCT (sTCT) was defined as the sequential addition of selexipag following a period of dual therapy with ERAs and PDE5is. In the sTCT strategy, only patients who received stable treatment with ERAs and PDE5is for at least 3 months were included. All patients were recommended to undergo a clinical evaluation every three months. The follow-up data were collected up to 23 March 2024 (follow-up cutoff time) to ensure adequate follow-up for evaluating the efficacy and safety of the therapy.

### Data collection

Non-invasive parameters were collected at baseline, 6 months, 12 months, and the last available follow-up. The baseline was established based on the clinical data obtained at the last clinical visit before the initiation of selexipag treatment. The collected data included the growth and development of patients; clinical manifestations of right heart failure, including exertional dyspnoea, right upper abdominal discomfort or pain, peripheral oedema, fatigue, nausea, and dizziness; history of syncope; WHO-FC; 6-min walk distance (6MWD); echocardiographic parameters, including pulmonary artery acceleration time (PAAT), right atrial diameter (RAD), right atrial area (RAA), tricuspid annular plane systolic excursion (TAPSE, in cm and z-score), right ventricular myocardial performance index (RVMPI), right ventricular diameter (RVD), and right ventricle/left ventricle (RV/LV) end-systolic diameter; as well as laboratory test results, including alanine aminotransferase (ALT), total bilirubin (TBIL), creatinine (Cr), haemoglobin (HGB), N-terminal pro-brain natriuretic peptide (NT-proBNP) and uric acid (UA) levels. Invasive haemodynamics were evaluated at baseline in all patients, with the decision to perform follow-up assessments made by the attending physician. Events, including cardiac surgery, discontinuation of selexipag, lung transplantation, and death, were observed. The follow-up endpoint was defined as the time to reach the cut-off date (23 March 2024), lung transplantation, or death. All events were recorded in patients who experienced multiple events.

### Titration of selexipag dosage

The initial dose for individualised treatment was determined by the physician. For children under 20 kg and over 20 kg, the usual initiating dose was 100 μg twice daily and 200 μg twice daily, respectively. The dose was recommended to further increase when the side effects subsided. An increase of 200 µg twice daily every week was recommended, but an increase of 200 μg once daily or 200 µg twice daily at longer intervals could also be considered, depending on the patient’s tolerance to side effects. The maximum dose was 1600 μg twice daily. If the patient is unable to tolerate the administered dose, appropriate dose reduction should be implemented. The maintenance dose was defined as the total daily dose received for the longest duration. Dose levels are defined as follows: low dose, a total of 400–800µg daily; medium dose, a total of 1000-2000µg daily; high dose, a total of 2200-3200μg daily.

### Statistical analysis

Analysis was performed using IBM SPSS Statistics 27.0, based on the baseline and follow-up clinical data, biomarker levels, and echocardiographic data. Descriptive statistics was used to summarise the demographic and clinical characteristics of the patients. Categorical data are presented as counts or percentages. Continuous and ordinal variables are presented as medians with interquartile ranges (IQRs). Paired Wilcoxon signed-rank tests were used for pairwise comparisons of baseline and follow-up values. A *P*-value < 0.05 was considered statistically significant (two-tailed test).

## Results

### Baseline characteristics

From 1 November 2018 to 30 September 2023, a total of 11 pediatric patients underwent initiation and titration of selexipag in our centre. One patient was excluded because the diagnosis was established by echocardiography rather RHC. Ultimately, 10 patients (7 females) were enrolled in the study. The demographic characteristics, diagnoses, and titration times of the patients are summarised in Table 1. Four patients (40%) were diagnosed with idiopathic PAH (IPAH), and four patients (40.0%) were diagnosed with hereditary PAH (HPAH), two of whom underwent genetic testing and both had *BMPR2* mutations. One patient’s mother had a history of PAH and one patient’s elder sister died of PAH. Two patients were diagnosed with congenital heart disease-related PAH (CHD-PAH): one had an isolated atrial septal defect (ASD), and the other had anomalous pulmonary venous connection (APVC) with concurrent ASD. All patients, except for one, were classified as WHO-FC II or III. Five patients underwent initial triple therapy, and another five patients received additional selexipag based on ERA and PDE5i (> 3 months) because dual use of ERA and PDE5i was unable to control disease progression and required escalated treatment. The median age at the start of selexipag titration was 14.5 (range 8.9–17.2) years.

**Table 1:** Baseline characteristics of the case series, presented as median (Q_1_, Q_3_) or count (%), *n* = 10

Demographics	
Age (years)	14.5 (12.1, 16.8)
Gender, female, n (%)	7 (70.0)
Height (cm)	157.5 (143.3, 166.8)
Weight (kg)	50.65 (33.13, 68.50)
BMI (kg/m^2^)	20.48 (16.80, 23.07)
BSA (m^2^)	1.49 (1.15, 1.73)
Clinical diagnosis	
PH Group 1	
1.1 IPAH	4 (40.0)
1.2 HPAH	4 (40.0)
1.4.4 CHD-PAH	2 (20.0)
Combination of selexipag, n	
Time from PAH diagnosis to selexipag initiation (months)	6.18 (0.18, 38.26)
Initial triple combination in newly diagnosed PAH	5 (50.0)
Third add-on to ERA and PDE5i combination	5 (50.0)
Time from PAH diagnosis to selexipag initiation (months)	26.8 (13.8,40.2)
Duration of treatment with selexipag (months)	23.25 (12.68,33.98)
Duration of follow-up (months)	29.3 (16.2,38.6)
WHO FC	
Class I	0 (0)
Class II	4 (40.0)
Class III	5 (50.0)
Class IV	1 (10.0)
Biomarker	
NT- proBNP (pg/ml)	837.2 (293.2, 1846.5)
6MWD (meters), *n* = 10	408.5 (315.8, 457.5)

*Abbreviations: 6MWD* 6-min walk distance, *BMI* body mass index, *BSA* body surface area, *CHD-PAH* congenital heart disease-related PAH, *ERA* endothelin receptor antagonist, *FC* functional class, *HPAH* hereditary PAH, *IPAH* idiopathic PAH, *NT-proBNP* N-terminal brain natriuretic peptide, *PH* pulmonary hypertension, *PAH* pulmonary arterial hypertension, *PDE-5i* phosphodiesterase-5 inhibitor, *WHO* World Health Organization

### Outcomes

The median follow-up time for the entire cohort was 29.3 (range 9.7–43.2) months. The median time from diagnosis to selexipag titration was 6.18 (range 0.1–83.0) months, and the median duration of selexipag therapy was 23.25 (range 9.7–43.2) months.

**Fig. 1:**
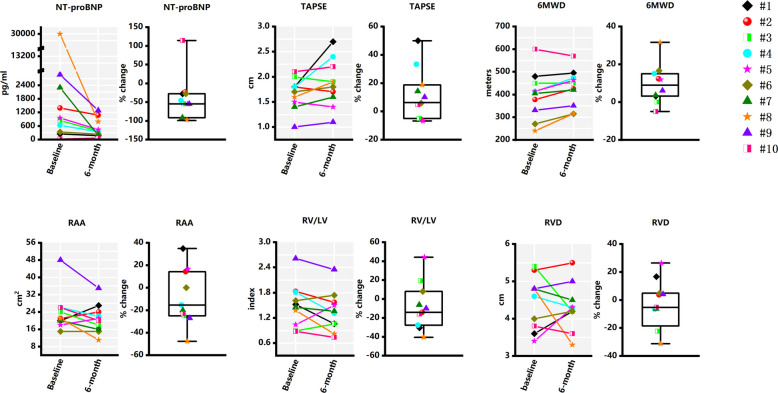
Changes in echocardiographic variables, 6MWD, and NT-proBNP at baseline and 6-month time point in all patients. It showed that most patients have a positive response to the treatment of selexipag. The box plot shows the median and quartiles, and the whiskers represent the minimum and maximum values. The scatter plot shows the percentage change in non-invasive variables for all patients. Abbreviation: NT-proBNP, N-terminal brain natriuretic peptide; RAD, right atrial diameter; RVD, right ventricular diameter; RV/LV, RV/LV end-systolic diameter; TAPSE, tricuspid annular plane systolic excursion; 6MWD, 6-min walk distance

Changes in NT-proBNP levels, TAPSE, 6MWD, and echocardiographic parameters from baseline to 6-month are illustrated in Fig. 1. At 6 months, most children showed improvements in NT-proBNP (*n* = 9, 90%) and 6MWD (*n* = 8, 80%). Favorable trends were also observed in echocardiographic parameters, including TAPSE and RAA. No deaths occurred during the first 6 months of follow-up.

**Fig. 2:**
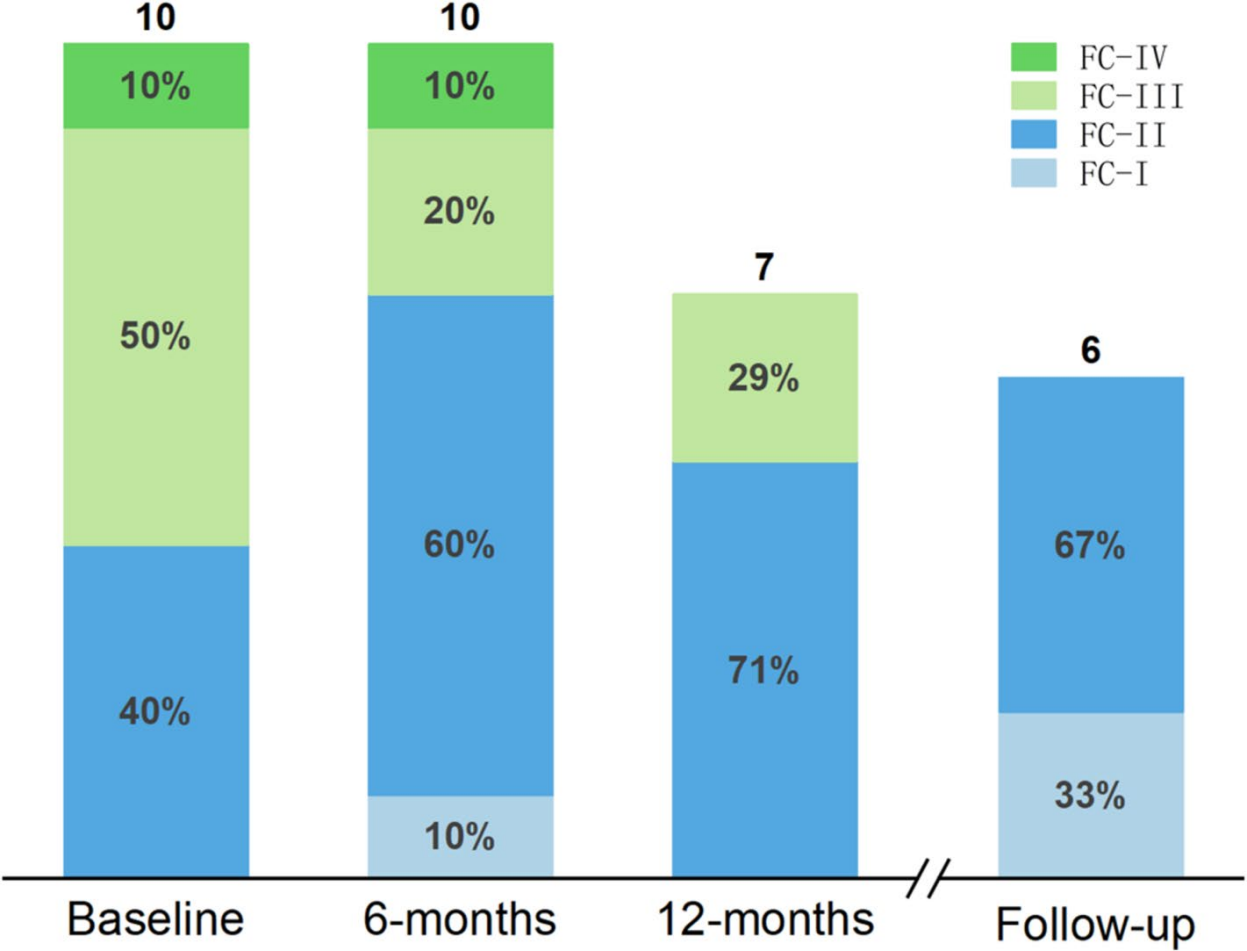
Changes in WHO functional class (FC) at baseline, 6-month, 12-month, and last follow-up time points in all 10 patients. WHO-FC was assessed at baseline, 6 months, and 12 months during scheduled clinical evaluations. For the last follow-up point, the most recent available WHO-FC prior to the cut-off date was recorded for all surviving patients

During the total follow-up period, 4 of 10 patients died. Paired comparisons were therefore performed in the 6 surviving patients with available follow-up data (Table 2). The WHO-FC improved in 5 patients and remained unchanged in 1 patient (*P* < 0.05). NT-proBNP levels decreased from a median of 587.6 (192.8, 8222.3) pg/ml to 181.7 (55.0, 553.4) pg/ml (*P* < 0.05). The median 6MWD increased by 70 m, although this did not reach statistical significance (*P* = 0.058). No statistically significant changes were observed in TAPSE or other echocardiographic parameters, including RAA, RVD, PAAT, RVMPI, and RV/LV end-systolic diameter. Changes in WHO-FC over time for all patients are presented in Fig. 2. Overall, WHO-FC improved after initiation of selexipag therapy in most patients during early follow-up, and this improvement was generally maintained among surviving patients at the last follow-up. At the cutoff date, all six surviving patients were classified as WHO-FC I (*n* = 2, 33.3%) or II (*n* = 4, 66.7%). Repeat cardiac catheterization data were available in two patients. Descriptive analyses demonstrated improvements in PVRI and cardiac function, as detailed in Additional file 1.

**Table 2:** Baseline and last follow-up characteristics of surviving patients (*n* = 6), presented as median (Q1, Q3)

	Baseline	Last follow-up	*P*-value
Growth and development			
Height (cm)	161.5 (152.5, 166.8)	167.5 (160.8, 173.5)	0.043
Weight (kg)	53.65(45.13, 68.50)	61.50 (53.00, 72.50)	0.075
BMI (kg/m^2^)	21.15 (17.48, 24.32)	21.90 (19.21, 25.26)	0.249
BSA (m^2^)	1.51 (1.40, 1.73)	1.65 (1.52, 1.84)	0.075
Echocardiographic variable			
RAD (cm)	4.55 (4.03, 5.25)	4.6 (3.7, 5.0)	0.753
RAA (cm^2^)	20.5 (17.3, 24.5)	20.0 (14.5, 21.8)	0.596
RVD (cm)	3.9 (3.6, 5.0)	4.0 (3.5, 4.2)	0.753
PAAT (ms)	63 (59, 96)	66 (61, 78)	1.000
RVMPI	0.50 (0.44, 0.58)	0.58 (0.41, 0.61)	0.599
RV/LV end-systolic diameter	1.21 (0.89, 1.54)	0.99 (0.72, 1.54)	0.345
TAPSE (cm)	1.8 (1.6, 2.0)	1.9 (1.4, 2.1)	1.000
TAPSE (z-score)	−2.76 (−3.95, −1.29)	−2.78 (−5.29, −1.10)	0.4000
Biomarker			
NT-proBNP (pg/ml)	587.6 (192.8, 8222.3)	181.7 (55.0, 553.4)	0.046
Uric acid (μmol/L)	403.5 (306.8, 608.0)	367.5 (272.3, 448.0)	0.249
Functional status			
WHO-FC	2.5 (2, 3)	2 (1, 2)	0.025
6MWD (m), *n*= 10	432.0 (262.5, 510.0)	502.0 (431.3, 566.3)	0.058

Differences between baseline and last follow-up data were analyzed using the Wilcoxon signed-rank test

*Abbreviations: BMI* body mass index, *BSA* body surface area, *FC* functional class, *LV* left ventricle, *m* meters, *MPI* myocardial performance index, *NT-proBNP* N-terminal brain natriuretic peptide, *PAAT* pulmonary artery acceleration time, *RAA* right atrial area, *RAD* right atrial diameter, *RV* right ventricle, *RVD* right ventricular diameter, *TAPSE* tricuspid annular plane systolic excursion, *WHO* World Health Organization, *6MWD* 6-min walk distance

A *P*-value < 0.05 was considered statistically significant (two-tailed test)

**Fig. 3:**
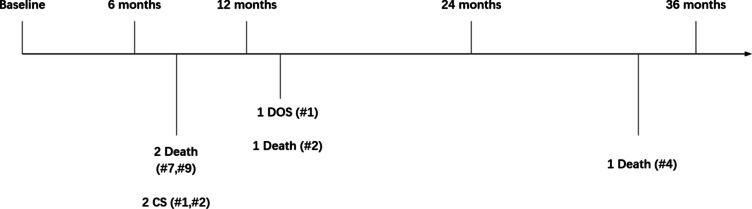
Timeline of all patients and the number of deaths, discontinuance of selexipag (DOS), and cardiac surgery (CS). Patients who discontinued selexipag (#1) were still followed up until the cut-off date. All events were recorded in patients who experienced multiple events

**Fig. 4:**
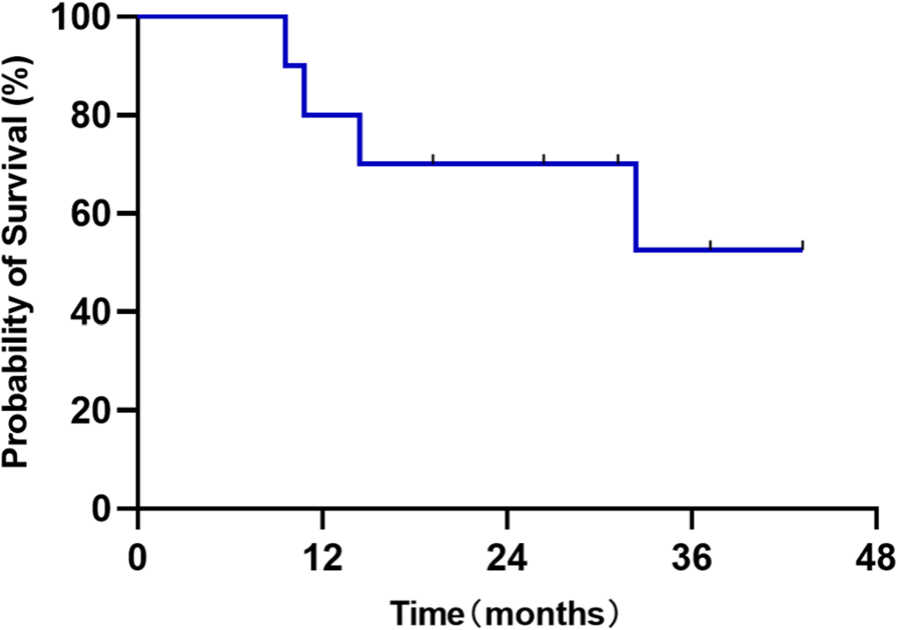
Kaplan–Meier overall survival curve for all 10 patients. The x-axis represents follow-up time in months, and the y-axis represents the probability of survival (%). Vertical tick marks on the curve indicate censored observations

Clinical events during follow-up are summarized in Fig. 3. During follow-up, 4 of 10 patients (40%) died. Transplant-free survival rates at 1, 2, and 3 years were 80%, 70%, and 60%, respectively. Kaplan–Meier survival curves are shown in Fig. 4.

### Tolerance, patient selection, and safety

All 10 patients underwent at least one titration and were titrated to a maintenance dose. The minimum body weight of children enrolled in our study was 25 kg, so none of them initiate at the lower dose level. The maintenance dose of the patients was mainly low dose (*n* = 4; 40.0%) and medium dose (*n* = 4; 40.0%), with a median of 1600 μg per day. Six patients experienced adverse reactions, including headaches (30.0%), loss of appetite and consequent weight loss (20%), jaw pain (10%), and increased bowel movements (10%). All adverse reactions were transient and did not result in selexipag discontinuation or dose reduction in any of the patients.

## Discussion

Paediatric PAH remains a fatal disease to date [5]. Based on current data, the use of PDE5i and ERA in combination early is recommended for patients at high risk; for patients who need escalated treatment, prostacyclin analogues and stimulators of prostacyclin receptors are added [8]. Intravenous or subcutaneous infusion of prostacyclin analogues increases the risk of infection and even death [23]. In China, treprostinil is not covered by medical insurance, which increases the economic burden [24]. Selexipag has been used as a substitute for prostacyclin analogues, with its effectiveness and safety in adults in China having been verified in our centre [25].

Since the first case of selexipag use in paediatric PAH was reported in 2017, large paediatric PH treatment centres in many countries have also reported their experiences [15, 16, 26]. A retrospective Canadian study described improvements in WHO-FC, 6MWD, and TAPSE, although these changes did not reach statistical significance, possibly due to limited sample size and heterogeneity in patient populations [19]. In a large multicenter retrospective cohort study including 87 patients, selexipag was associated with early improvements in invasive hemodynamic parameters, particularly a reduction in the pulmonary-to-systemic vascular resistance ratio (PVRi/SVRi) in patients receiving add-on therapy. However, patients transitioned from other prostanoids showed relative hemodynamic stability but experienced late functional decline in some cases, underscoring the complexity of treatment response and the need for longitudinal monitoring [26].

In comparison, our study primarily evaluated non-invasive parameters that are more commonly used in routine pediatric follow-up. We observed improvements in WHO FC, NT-proBNP, and 6-min walk distance, particularly within the first 6 months of therapy. Although haemodynamic data are limited (from 2 patients), these findings appear broadly consistent with early improvements reported in multicentre cohorts. However, it should be noted that previously published studies have included heterogeneous populations, such as patients receiving add-on therapy or those undergoing treatment transition. In contrast, our study specifically focused on children receiving triple combination therapy, which may represent a more intensive treatment strategy.

All adverse reactions were transient, with the most common being headache (3/10), consistent with the results of the study on Chinese adults in our centre. Although multiple studies have reported vomiting as the most frequent adverse event, the most common ones in practice are still gastrointestinal disturbances (including vomiting, diarrhea, and constipation) and headache [20, 26]. These reactions are generally transient, and no deaths have been directly attributed to the use of selexipag.

In our study, patients were empirically stratified by body weight (< 20 kg and ≥ 20 kg), with selexipag initiated at 100 μg or 200 μg twice daily and titrated to target doses of 800 μg or 1,600 μg twice daily, respectively. This weight-based approach appeared to be associated with improved outcomes in pediatric PAH. In contrast, Axelsen et al. applied age- and weight-based stratification and demonstrated comparable pharmacokinetic profiles of selexipag and its active metabolite between pediatric and adult populations [27], an approach subsequently adopted in several recent phase II pediatric studies [21, 26]. Although absolute doses differed, all titration strategies emphasized careful escalation adjusted for age and weight, highlighting the need for standardized pediatric protocols. The link between target dose and outcomes or prognosis also warrants further investigation.

This study has several limitations. Firstly, This was a retrospective, single-centre study with a small sample size, potentially leading to selection bias and limited statistical power and generalizability. As the limited sample size did not allow for subgroup adjustment, heterogeneity in patient characteristics and treatment strategies may have confounded the observed outcomes. Secondly, efficacy assessment relied predominantly on non-invasive parameters, while invasive haemodynamic data were limited. Thirdly, the absence of a control group precludes causal inference. Finally, dosing strategies were not standardised and pharmacokinetic data were unavailable, limiting interpretation of dose–response relationships.

## Conclusion

Our real-world data suggest that selexipag, as part of triple combination therapy, may provide clinical benefits in children with PAH. In addition to improvements in functional status and biomarker profiles during early follow-up, sustained favorable prognostic outcomes were achieved in patients with mild disease over long-term follow-up, with an acceptable safety profile. Nevertheless, larger prospective multicentre studies with integrated haemodynamic and pharmacokinetic assessments are required to confirm these observations and to optimise treatment strategies in this population.

## Supplementary Information

Supplementary Material 1.

Supplementary Material 2.

Supplementary Material 3.

## Data Availability

All data generated or analysed during this study are included in this published article and its Supplementary Information. The anonymized individual-level data supporting the findings of this study are provided in the Supplementary Information (Additional file 2 and Additional file 3).

## Abbreviations

6MWD: 6-Minute walk distance
ALT: Alanine aminotransferase
APVC: Anomalous pulmonary venous connection
ASD: Atrial septal defect
CHD-PAH: Congenital heart disease-related PAH
Cr: Creatinine
DPG: Diastolic transpulmonary pressure gradient
EPPVDN: European Paediatric Pulmonary Vascular Disease Network
ERAs: Endothelial receptor antagonists
FC: Functional class
HGB: Haemoglobin
HPAH: Hereditary PAH
IPAH: Idiopathic PAH
IQRs: Interquartile ranges
LVEDP: Left ventricular end-diastolic pressure
mPAP: Mean pulmonary artery pressure
NT-proBNP: N-terminal pro-brain natriuretic peptide
PAAT: Pulmonary artery acceleration time
PAH: Pulmonary arterial hypertension
PAP: Pulmonary artery pressure
PAWP: Pulmonary artery wedge pressure
PDE5is: Phosphodiesterase-5 inhibitors
PH: Pulmonary hypertension
PVRI: Pulmonary vascular resistance index
PVRi/SVRi: Pulmonary to systemic vascular resistance ratio
RAA: Right atrial area
RAD: Right atrial diameter
RHC: Right heart catheterisation
RV/LV: Right ventricle/left ventricle
RVD: Right ventricular diameter
RVMPI: Right ventricular myocardial performance index
S/D: Systolic-to-diastolic duration ratio
sPAP: Systolic pulmonary artery pressure
sTCT: Sequential TCT
TAPSE: Tricuspid annular plane systolic excursion
TBIL: Total bilirubin
TCT: Triple combination therapy
TRV: Tricuspid regurgitation velocity
UA: Uric acid
uTCT: Upfront TCT
WHO-FC: WHO functional class
WSPH: World Symposium on Pulmonary Hypertension
