# Supplementary material for: Experience with selexipag in triple therapy for pulmonary arterial hypertension in Chinese children
Source: BMC Pediatr. 2026 May 8;26:601. doi: 10.1186/s12887-026-06954-9 (PMC13325776; doi:10.1186/s12887-026-06954-9)
Supplement: Supplementary file 1 — Supplementary Material 1. [file 12887_2026_6954_MOESM1_ESM.docx]

**Title**: Experience with Selexipag in Triple Therapy for Pulmonary Arterial Hypertension in Chinese Children.

**Journal**: BMC Pediatrics

**Authors**: Meng Li, Yingchun Wang, Xiaoyu Hu, Haizhao Zhao, Weida Lu, Yuan Ji, and Xiaopei Cui

**Corresponding Author:**

Xiaopei Cui, MD, PhD

Affiliation: Department of Geriatric Medicine & Laboratory of Gerontology and Anti-Aging Research, Qilu Hospital, Cheeloo College of Medicine, Shandong University, No.107 West Wenhua Road, Jinan, 250012, Shandong Province, China

Email: cuixiaopei@sdu.edu.cn

**Table S1. Hemodynamic changes in patients with follow-up cardiac catheterization.**

| Parameter | **#2** Baseline | **#2** Follow-up  (6-month) | **#3** Baseline | **#3** Follow-up  (40.8-month) |
| --- | --- | --- | --- | --- |
| mPAP (mmHg) | 85 | 65 | 76 | 76 |
| mRAP (mmHg) | 5 | 5 | 3 | 3 |
| PAWP (mmHg) | 7 | 7 | 10 | 7 |
| CI (L/min/m²) | 3.58 | 3.23 | 2.06 | 3.37 |
| PVRI (WU·m²) | 16.80 | 13.37 | 30.20 | 22.56 |
| SvO₂ (%) | 59.25 | 64.50 | 63.30 | 67.75 |
| SaO₂ (%) | 77 | 92 | 81 | 97 |
| Vasoreactivity | Negative | Negative | Negative | Negative |

Data are presented as individual values. Due to the limited number of patients (n=2) and heterogeneous follow-up durations, no statistical analysis was performed. Abbreviations: CI, cardiac index; mPAP, mean pulmonary arterial pressure; mRAP, mean right atrial pressure; PAWP, pulmonary arterial wedge pressure; PVRI, pulmonary vascular resistance index; SaO2, arterial oxygen saturation ; SvO2, venous oxygen saturation.
