# Supplementary material for: Experience with selexipag in triple therapy for pulmonary arterial hypertension in Chinese children
Source: BMC Pediatr. 2026 May 8;26:601. doi: 10.1186/s12887-026-06954-9 (PMC13325776; doi:10.1186/s12887-026-06954-9)
Supplement: Supplementary file 2 — Supplementary Material 2. [file 12887_2026_6954_MOESM2_ESM.docx]

**Title**: Experience with Selexipag in Triple Therapy for Pulmonary Arterial Hypertension in Chinese Children.

**Journal**: BMC Pediatrics

**Authors**: Meng Li, Yingchun Wang, Xiaoyu Hu, Haizhao Zhao, Weida Lu, Yuan Ji, and Xiaopei Cui

**Corresponding Author:**

Xiaopei Cui, MD, PhD

Affiliation: Department of Geriatric Medicine & Laboratory of Gerontology and Anti-Aging Research, Qilu Hospital, Cheeloo College of Medicine, Shandong University, No.107 West Wenhua Road, Jinan, 250012, Shandong Province, China

Email: cuixiaopei@sdu.edu.cn

**Table S2.** **Individual PAH patient characteristics at the time of baseline and medication of selexipag.**

| **ID** | **Gender (M/F)** | **Age (y)** | **BMI (kg/m²）** | **PAH etiology** | **FC** | **NT-proBNP (pg/mL)** | **subsequent RHC** | **Medication of selexipag** | | | | **Duration of follow-up**  **(weeks)** | **Outcome** |
| --- | --- | --- | --- | --- | --- | --- | --- | --- | --- | --- | --- | --- | --- |
|  |  |  |  |  |  |  |  | **uTCT/**  **sTCT** | **Time from diagnosis (months)** | **1^st^ dose (μg)**  **(Interval weeks)**  **Maintenance dose (µg)** | **Duration of selexipag treatment**  **(months)** |  |  |
| 1 | F | 15.9 | 18.00 | 1.4.4  CHD-PAH | Ⅲ | 248.3 | No | uTCT | 0.33 | 400 (12.4) 1600 | 13.2 | 42.9 | Alive |
| 2 | F | 12.9 | 16.80 | 1.4.4  CHD-PAH | Ⅲ | 1400.0 | Yes | sTCT | 43.43 | 400 (36.3) 3200 | 17.9 | 17.9 | Death |
| 3 | M | 12.7 | 23.31 | 1.2  HPAH | Ⅱ | 837.2 | Yes | sTCT | 12.03 | 400 (151.7) 3200 | 43.2 | 43.2 | Alive |
| 4 | F | 8.9 | 16.80 | 1.1  IPAH | Ⅲ | 628.3 | No | uTCT | 0.10 | 400 (12.8) 600 | 32.9 | 32.9 | Death |
| 5 | F | 10.2 | 15.93 | 1.2  HPAH | Ⅱ | 963.0 | No | uTCT | 0.13 | 400 (13.4) 800 | 19.7 | 19.7 | Alive |
| 6 | F | 13.0 | 19.31 | 1.1  IPAH | Ⅲ | 338.0 | No | sTCT | 83.00 | 400 (38.3) 1600 | 37.2 | 37.2 | Alive |
| 7 | M | 17.2 | 22.09 | 1.1  IPAH | Ⅱ | 2293.0 | No | uTCT | 0.33 | 400 (260) 2000 | 11.1 | 11.1 | Death |
| 8 | F | 16.7 | 27.34 | 1.2  HPAH | Ⅲ | >30000.0 | No | uTCT | 0.20 | 400 (16.8) 800 | 31.8 | 31.8 | Alive |
| 9 | F | 16.9 | 21.64 | 1.2  HPAH | Ⅳ | 2869.0 | No | sTCT | 36.53 | 400  (6.5) 800 | 9.7 | 9.7 | Death |
| 10 | M | 16.0 | 22.99 | 1.1  IPAH | Ⅱ | 26.4 | No | sTCT | 13.20 | 400 (5.1) 3200 | 26.8 | 26.8 | Alive |

UTCT, upfront triple combination therapy, was defined as patients who initiated triple therapy including selexipag, ERAs, and PDE5is within 6 weeks after diagnosis. STCT, sequential triple combination therapy, was defined as the sequential addition of selexipag following a period of dual therapy with ERAs and PDE5is. In the sTCT strategy, all patients received stable treatment with ERAs and DE5i for at least 3 months. 1^st^ dose, Initial titration dose. Maintenance dose, the total daily dose received for the longest duration. One patient (#1) discontinued selexipag before cut-off date, who orally administered macitentan for monotherapy at 1.1 years. Abbreviations: BMI: body mass index; CHD-PAH, congenital heart disease-related PAH; F, female; FC, function class; HPAH, hereditary PAH; IPAH, idiopathic PAH; M, male; NT-proBNP, N-terminal pro-brain natriuretic peptide; PAH, pulmonary arterial hypertension; RHC, right heart catheterization; y, years.
