# Supplementary material for: Experience with selexipag in triple therapy for pulmonary arterial hypertension in Chinese children
Source: BMC Pediatr. 2026 May 8;26:601. doi: 10.1186/s12887-026-06954-9 (PMC13325776; doi:10.1186/s12887-026-06954-9)
Supplement: Supplementary file 3 — Supplementary Material 3. [file 12887_2026_6954_MOESM3_ESM.docx]

**Title**: Experience with Selexipag in Triple Therapy for Pulmonary Arterial Hypertension in Chinese Children.

**Journal**: BMC Pediatrics

**Authors**: Meng Li, Yingchun Wang, Xiaoyu Hu, Haizhao Zhao, Weida Lu, Yuan Ji, and Xiaopei Cui

**Corresponding Author:**

Xiaopei Cui, MD, PhD

Affiliation: Department of Geriatric Medicine & Laboratory of Gerontology and Anti-Aging Research, Qilu Hospital, Cheeloo College of Medicine, Shandong University, No.107 West Wenhua Road, Jinan, 250012, Shandong Province, China

Email: cuixiaopei@sdu.edu.cn

**Table S3. Individual 10 PAH patients’ (#1-#10） demographics，echocardiographic parameters, laboratory test results medication，and functional status at the time of baseline, 6-month, 12-month and last follow up.**

**#1**

|  | **Baseline** | **6-month** | **12-month** | **Last follow-up** |
| --- | --- | --- | --- | --- |
| **Demographics** | | | | |
| Age (years) | 15.9 | 16.4 | 16.9 | 16.9 |
| Height（cm） | 165.0 | 165.0 | 165.0 | 165.0 |
| Weight（kg） | 49.00 | 51.00 | 57.00 | 57.00 |
| BMI（kg/m²） | 18.00 | 18.73 | 20.94 | 20.94 |
| BSA（m²） | 1.48 | 1.50 | 1.58 | 1.58 |
| **Echocardiography (M-mode）** | | | | |
| RAD（cm）,PSAX | 3.8 | 5.3 | 3.4 | 3.4 |
| RAA（cm²）,PSAX | 20 | 27 | 15 | 15 |
| RVD (cm), PSAX | 3.6 | 4.2 | 4.0 | 4.0 |
| TAPSE（cm） | 1.8 (z -2.65) | 2.7 (z 1.59) | 1.4 (z -4.83) | 1.4 (z -4.83) |
| PAAT（ms）,PSAX | 102 | 91 | 63 | 63 |
| LVEF (%) | 79 | 65 | 60 | 60 |
| RVMPI | 0.63 | 0.64 | 0.59 | 0.59 |
| Pericardial effusion | Yes | No | No | No |
| RV/LV | 1.52 | 1.06 | 0.65 | 0.65 |
| **Laboratory test** | | | | |
| NT-proBNP (pg/ml) | 248.3 | 180.0 | 50.2 | 50.2 |
| ALT (U/L) | 9.0 | 7.0 | 9.0 | 9.0 |
| TBIL (μmol/L) | 9.3 | 8.7 | 9.4 | 9.4 |
| Cr (μmol/L) | 80.0 | 77.0 | 60.0 | 60.0 |
| UA (μmol/L) | 380.0 | 277.0 | 192.0 | 192.0 |
| HGB (g/L) | 126.0 | 116.0 | 119.0 | 119.0 |
| **Functional status** | | | | |
| WHO FC | Ⅲ | Ⅱ | Ⅱ | Ⅱ |
| 6MWD (m） | 480 | 495 | 565 | 565 |

Abbreviations: 6MWD, 6-minute walk distance; ALT, alanine aminotransferase; BMI, body mass index; BSA, body surface area; CI, cardiac index; Cr, creatinine; FC, functional class; HGB, haemoglobin; LVEF, left ventricular ejection fraction; MPI, myocardial performance index; NT-proBNP, N-terminal brain natriuretic peptide; PAH, pulmonary arterial hypertension; PAAT, pulmonary artery acceleration time; RAA, right atrial area; RAD, right atrial diameter; RV, right ventricle; RVD, right ventricular diameter; RV/LV, RV/LV end-systolic diameter; TAPSE, tricuspid annular plane systolic excursion; TBIL, total bilirubin; UA, uric acid; WHO, World Health Organization.

**#2**

|  | **Baseline** | **6-month** | **12-month** | **Last follow-up** |
| --- | --- | --- | --- | --- |
| **Demographics** | | | | |
| Age (years) | 12.9 | 13.4 | 13.9 | 14.3 |
| Height（cm） | 138.0 | 145.0 | 151.0 | 152.0 |
| Weight（kg） | 32.00 | 36.00 | 42.00 | 42.50 |
| BMI（kg/m²） | 16.80 | 17.12 | 18.42 | 18.40 |
| BSA（m²） | 1.10 | 1.19 | 1.31 | 1.32 |
| **Echocardiography (M-mode）** | | | | |
| RAD（cm）,PSAX | 5.4 | 5.2 | 5.0 | 5.6 |
| RAA（cm²）,PSAX | 21 | 24 | 23 | 28 |
| RVD (cm), PSAX | 5.3 | 5.5 | 5.6 | 5.6 |
| TAPSE（cm） | 1.8 (z -2.27） | 1.7 (z -2.86) | 2.0 (z -1.14) | 2.3 (z -0.21) |
| PAAT（ms）,PSAX | 68 | 54 | 55 | 55 |
| LVEF (%) | 60 | 60 | 60 | 60 |
| RVMPI | 0.41 | 0.54 | 0.52 | 0.37 |
| Pericardial effusion | No | No | No | No |
| RV/LV | 1.83 | 1.57 | 1.69 | 2.00 |
| **Laboratory test** | | | | |
| NT-proBNP (pg/ml) | 1400.0 | 1080.0 | 2436.0 | 1491.0 |
| ALT (U/L) | 14.0 | 8.0 | 17.0 | 13.0 |
| TBIL (μmol/L) | 8.9 | 15.8 | 17.9 | 12.3 |
| Cr (μmol/L) | 81.0 | 51.0 | 89.0 | 66.0 |
| UA (μmol/L) | 245.0 | 318.0 | 278.0 | 249.0 |
| HGB (g/L) | 119.0 | 123.0 | 113.0 | 131.0 |
| **Functional status** | | | | |
| WHO FC | Ⅲ | Ⅱ | Ⅲ | Ⅲ |
| 6MWD (m） | 378 | 425 | 395 | 480 |

Abbreviations: 6MWD, 6-minute walk distance; ALT, alanine aminotransferase; BMI, body mass index; BSA, body surface area; CI, cardiac index; Cr, creatinine; FC, functional class; HGB, haemoglobin; LVEF, left ventricular ejection fraction; MPI, myocardial performance index; NT-proBNP, N-terminal brain natriuretic peptide; PAH, pulmonary arterial hypertension; PAAT, pulmonary artery acceleration time; RAA, right atrial area; RAD, right atrial diameter; RV, right ventricle; RVD, right ventricular diameter; RV/LV, RV/LV end-systolic diameter; TAPSE, tricuspid annular plane systolic excursion; TBIL, total bilirubin; UA, uric acid; WHO, World Health Organization.

**#3**

|  | **Baseline** | **6-month** | **12-month** | **Last follow-up** |
| --- | --- | --- | --- | --- |
| **Demographics** | | | | |
| Age (years) | 12.7 | 13.2 | 13.7 | 16.1 |
| Height（cm） | 155.0 | 160.0 | 167.0 | 173.0 |
| Weight（kg） | 56.00 | 60.00 | 69.00 | 80.00 |
| BMI（kg/m²） | 23.31 | 23.44 | 24.74 | 26.73 |
| BSA（m²） | 1.51 | 1.59 | 1.75 | 1.93 |
| **Echocardiography (M-mode）** | | | | |
| RAD（cm）,PSAX | 5.4 | 4.3 | 4.5 | 4.8 |
| RAA（cm²）,PSAX | 24 | 18 | 19 | 20 |
| RVD (cm), PSAX | 5.4 | 4.2 | 4.1 | 3.9 |
| TAPSE（cm） | 2.0 (z -0.93) | 1.9 (z -1.71) | 2.0 (z -1.14) | 2.1 (z -1.41) |
| PAAT（ms）,PSAX | 56 | 67 | 55 | 66 |
| LVEF (%) | 65 | 60 | 60 | 60 |
| RVMPI | 0.56 | 0.54 | 0.54 | 0.41 |
| Pericardial effusion | Yes | No | No | No |
| RV/LV | 0.89 | 1.06 | 1.38 | 0.91 |
| **Laboratory test** | | | | |
| NT-proBNP (pg/ml) | 837.2 | 382.2 | 238.3 | 127.2 |
| ALT (U/L) | 27.0 | 20.0 | 17.0 | 26.0 |
| TBIL (μmol/L) | 12.7 | 10.9 | 14.9 | 13.1 |
| Cr (μmol/L) | 46.0 | 54.0 | 47.0 | 61.0 |
| UA (μmol/L) | 629.0 | 444.0 | 702.0 | 427.0 |
| HGB (g/L) | 121.0 | 129.0 | 128.0 | 167.0 |
| **Functional status** | | | | |
| WHO FC | Ⅱ | Ⅱ | Ⅱ | Ⅰ |
| 6MWD (m） | 450 | 450 | 438 | 480 |

Abbreviations: 6MWD, 6-minute walk distance; ALT, alanine aminotransferase; BMI, body mass index; BSA, body surface area; CI, cardiac index; Cr, creatinine; FC, functional class; HGB, haemoglobin; LVEF, left ventricular ejection fraction; MPI, myocardial performance index; NT-proBNP, N-terminal brain natriuretic peptide; PAH, pulmonary arterial hypertension; PAAT, pulmonary artery acceleration time; RAA, right atrial area; RAD, right atrial diameter; RV, right ventricle; RVD, right ventricular diameter; RV/LV, RV/LV end-systolic diameter; TAPSE, tricuspid annular plane systolic excursion; TBIL, total bilirubin; UA, uric acid; WHO, World Health Organization.

**#4**

|  | **Baseline** | **6-month** | **12-month** | **Last follow-up** |
| --- | --- | --- | --- | --- |
| **Demographics** | | | | |
| Age (years) | 8.9 | 9.4 | 9.9 | 10.8 |
| Height（cm） | 122.0 | 123.0 | 126.0 | 130.0 |
| Weight（kg） | 25.00 | 25.00 | 30.00 | 33.00 |
| BMI（kg/m²） | 16.80 | 16.52 | 18.90 | 19.53 |
| BSA（m²） | 0.91 | 0.92 | 0.94 | 0.96 |
| **Echocardiography (M-mode）** | | | | |
| RAD（cm）,PSAX | 6.0 | 4.7 | 3.9 | 4.1 |
| RAA（cm²）,PSAX | 26 | 22 | 13 | 15 |
| RVD (cm), PSAX | 4.6 | 4.3 | 3.3 | 3.4 |
| TAPSE（cm） | 1.8 (z -1.13) | 2.4 (z 2.69) | 2.0 (z -0.07) | 1.9 (z -1.15) |
| PAAT（ms）,PSAX | 68 | 54 | 74 | 66 |
| LVEF (%) | 55 | 60 | 60 | 60 |
| RVMPI | 0.41 | 0.38 | 0.34 | 0.55 |
| Pericardial effusion | No | No | No | No |
| RV/LV | 1.81 | 1.31 | 0.65 | 0.84 |
| **Laboratory test** | | | | |
| NT-proBNP (pg/ml) | 628.3 | 341.0 | 181.4 | 502.8 |
| ALT (U/L) | 9.0 | 17.0 | 10.0 | 8.0 |
| TBIL (μmol/L) | 8.7 | 6.6 | 5.8 | 3.9 |
| Cr (μmol/L) | 63.0 | 32.0 | 31.0 | 41.0 |
| UA (μmol/L) | 189.0 | 214.0 | 271.0 | 191.0 |
| HGB (g/L) | 156.0 | 132.0 | 113.0 | 139.0 |
| **Functional status** | | | | |
| WHO FC | Ⅲ | Ⅱ | Ⅱ | Ⅱ |
| 6MWD (m） | 412 | 474 | 443 | 540 |

Abbreviations: 6MWD, 6-minute walk distance; ALT, alanine aminotransferase; BMI, body mass index; BSA, body surface area; CI, cardiac index; Cr, creatinine; FC, functional class; HGB, haemoglobin; LVEF, left ventricular ejection fraction; MPI, myocardial performance index; NT-proBNP, N-terminal brain natriuretic peptide; PAH, pulmonary arterial hypertension; PAAT, pulmonary artery acceleration time; RAA, right atrial area; RAD, right atrial diameter; RV, right ventricle; RVD, right ventricular diameter; RV/LV, RV/LV end-systolic diameter; TAPSE, tricuspid annular plane systolic excursion; TBIL, total bilirubin; UA, uric acid; WHO, World Health Organization.

**#5**

|  | **Baseline** | **6-month** | **12-month** | **Last follow-up** |
| --- | --- | --- | --- | --- |
| **Demographics** | | | | |
| Age (years) | 10.2 | 10.7 | 11.2 | 11.7 |
| Height（cm） | 145.0 | 152.0 | 154.0 | 157.0 |
| Weight（kg） | 33.50 | 37.00 | 37.40 | 41.00 |
| BMI（kg/m²） | 15.93 | 16.01 | 15.77 | 16.63 |
| BSA（m²） | 1.16 | 1.25 | 1.27 | 1.33 |
| **Echocardiography (M-mode）** | | | | |
| RAD（cm）,PSAX | 4.3 | 4.9 | 5.1 | 5.4 |
| RAA（cm²）,PSAX | 18 | 21 | 22 | 24 |
| RVD (cm), PSAX | 3.4 | 4.3 | 4.6 | 4.1 |
| TAPSE（cm） | 1.5 (z -4.23） | 1.4 (z -5.00) | 1.7 (z -2.96) | 1.2 (z -6.67) |
| PAAT（ms）,PSAX | 63 | 45 | 40 | 54 |
| LVEF (%) | 60 | 60 | 60 | 60 |
| RVMPI | 0.55 | 0.59 | 0.71 | 0.67 |
| Pericardial effusion | No | No | Yes | Yes |
| RV/LV | 1.04 | 1.50 | 1.64 | 1.79 |
| **Laboratory test** | | | | |
| NT-proBNP (pg/ml) | 963.0 | 435.0 | 390.9 | 532.6 |
| ALT (U/L) | 14.0 | 18.0 | 22.0 | 11.0 |
| TBIL (μmol/L) | 8.3 | 12.0 | 6.2 | 10.8 |
| Cr (μmol/L) | 41.0 | 41.0 | 49.0 | 50.0 |
| UA (μmol/L) | 427.0 | 422.0 | 413.0 | 447.0 |
| HGB (g/L) | 138.0 | 145.0 | 131.0 | 149.0 |
| **Functional status** | | | | |
| WHO FC | Ⅱ | Ⅱ | Ⅱ | Ⅱ |
| 6MWD (m） | 414 | 463 | 482 | 524 |

Abbreviations: 6MWD, 6-minute walk distance; ALT, alanine aminotransferase; BMI, body mass index; BSA, body surface area; CI, cardiac index; Cr, creatinine; FC, functional class; HGB, haemoglobin; LVEF, left ventricular ejection fraction; MPI, myocardial performance index; NT-proBNP, N-terminal brain natriuretic peptide; PAH, pulmonary arterial hypertension; PAAT, pulmonary artery acceleration time; RAA, right atrial area; RAD, right atrial diameter; RV, right ventricle; RVD, right ventricular diameter; RV/LV, RV/LV end-systolic diameter; TAPSE, tricuspid annular plane systolic excursion; TBIL, total bilirubin; UA, uric acid; WHO, World Health Organization.

**#6**

|  | **Baseline** | **6-month** | **12-month** | **Last follow-up** |
| --- | --- | --- | --- | --- |
| **Demographics** | | | | |
| Age (years) | 13.0 | 13.5 | 14.0 | 15.5 |
| Height（cm） | 163.0 | 167.0 | 168.0 | 170.0 |
| Weight（kg） | 51.30 | 57.50 | 57.00 | 58.00 |
| BMI（kg/m²） | 19.31 | 20.62 | 20.20 | 20.07 |
| BSA（m²） | 1.50 | 1.60 | 1.60 | 1.63 |
| **Echocardiography (M-mode）** | | | | |
| RAD（cm）,PSAX | 4.1 | 4.2 | 4.2 | 4.8 |
| RAA（cm²）,PSAX | 15 | 15 | 16 | 21 |
| RVD (cm), PSAX | 4.0 | 4.2 | 4.3 | 4.6 |
| TAPSE（cm） | 1.7 (z -2.86) | 1.8 (z -2.29) | 2.0 (z-1.33) | 2.1 (z -1.15) |
| PAAT（ms）,PSAX | 60 | 51 | 74 | 66 |
| LVEF (%) | 60 | 60 | 60 | 60 |
| RVMPI | 0.45 | 0.45 | 0.60 | 0.58 |
| Pericardial effusion | No | No | No | No |
| RV/LV | 1.61 | 1.74 | 1.23 | 1.46 |
| **Laboratory test** | | | | |
| NT-proBNP (pg/ml) | 338.0 | 244.0 | 161.7 | 236.2 |
| ALT (U/L) | 10.0 | 11.0 | 7.0 | 8.0 |
| TBIL (μmol/L) | 6.0 | 13.9 | 9.8 | 11.0 |
| Cr (μmol/L) | 53.0 | 52.0 | 48.0 | 55.0 |
| UA (μmol/L) | 319.0 | 323.0 | 348.0 | 308.0 |
| HGB (g/L) | 143.0 | 132.0 | 137.0 | 123.0 |
| **Functional status** | | | | |
| WHO FC | Ⅲ | Ⅲ | Ⅲ | Ⅱ |
| 6MWD (m） | 270 | 315 | 300 | 420 |

Abbreviations: 6MWD, 6-minute walk distance; ALT, alanine aminotransferase; BMI, body mass index; BSA, body surface area; CI, cardiac index; Cr, creatinine; FC, functional class; HGB, haemoglobin; LVEF, left ventricular ejection fraction; MPI, myocardial performance index; NT-proBNP, N-terminal brain natriuretic peptide; PAH, pulmonary arterial hypertension; PAAT, pulmonary artery acceleration time; RAA, right atrial area; RAD, right atrial diameter; RV, right ventricle; RVD, right ventricular diameter; RV/LV, RV/LV end-systolic diameter; TAPSE, tricuspid annular plane systolic excursion; TBIL, total bilirubin; UA, uric acid; WHO, World Health Organization.

**#7**

|  | **Baseline** | **6-month** | **12-month** | **Last follow-up** |
| --- | --- | --- | --- | --- |
| **Demographics** | | | | |
| Age (years) | 17.2 | 17.7 |  | 17.7 |
| Height（cm） | 178.0 | 178.0 |  | 178.0 |
| Weight（kg） | 70.00 | 68.00 |  | 68.00 |
| BMI（kg/m²） | 22.09 | 21.46 |  | 21.46 |
| BSA（m²） | 1.83 | 1.80 |  | 1.80 |
| **Echocardiography (M-mode）** | | | | |
| RAD（cm）,PSAX | 4.4 | 3.9 |  | 3.9 |
| RAA（cm²）,PSAX | 20 | 16 |  | 16 |
| RVD (cm), PSAX | 4.8 | 4.5 |  | 4.5 |
| TAPSE（cm） | 1.4 (z -5.12) | 1.6 (z -4.15) |  | 1.6 (z -4.15) |
| PAAT（ms）,PSAX | 64 | 54 |  | 54 |
| LVEF (%) | 50 | 50 |  | 50 |
| RVMPI | 0.72 | 0.50 |  | 0.50 |
| Pericardial effusion | Yes | No |  | No |
| RV/LV | 1.45 | 1.36 |  | 1.36 |
| **Laboratory test** | | | | |
| NT-proBNP (pg/ml) | 2293.0 | 194.0 |  | 194.0 |
| ALT (U/L) | 14.0 | 17.0 |  | 17.0 |
| TBIL (μmol/L) | 10.2 | 6.8 |  | 6.8 |
| Cr (μmol/L) | 91.0 | 98.0 |  | 98.0 |
| UA (μmol/L) | 589.0 | 251.0 |  | 251.0 |
| HGB (g/L) | 142.0 | 136.0 |  | 136.0 |
| **Functional status** | | | | |
| WHO FC | Ⅱ | Ⅱ |  | Ⅱ |
| 6MWD (m） | 405 | 420 |  | 420 |

Abbreviations: 6MWD, 6-minute walk distance; ALT, alanine aminotransferase; BMI, body mass index; BSA, body surface area; CI, cardiac index; Cr, creatinine; FC, functional class; HGB, haemoglobin; LVEF, left ventricular ejection fraction; MPI, myocardial performance index; NT-proBNP, N-terminal brain natriuretic peptide; PAH, pulmonary arterial hypertension; PAAT, pulmonary artery acceleration time; RAA, right atrial area; RAD, right atrial diameter; RV, right ventricle; RVD, right ventricular diameter; RV/LV, RV/LV end-systolic diameter; TAPSE, tricuspid annular plane systolic excursion; TBIL, total bilirubin; UA, uric acid; WHO, World Health Organization.

**#8**

|  | **Baseline** | **6-month** | **12-month** | **Last follow-up** |
| --- | --- | --- | --- | --- |
| **Demographics** | | | | |
| Age (years) | 16.7 | 17.2 | 17.7 | 17.7 |
| Height（cm） | 160.0 | 162.0 | 162.0 | 162.0 |
| Weight（kg） | 70.00 | 69.00 | 65.00 | 65.00 |
| BMI（kg/m²） | 27.34 | 26.29 | 24.77 | 24.77 |
| BSA（m²） | 1.72 | 1.72 | 1.67 | 1.67 |
| **Echocardiography (M-mode）** | | | | |
| RAD（cm）,PSAX | 4.8 | 3.6 | 3.8 | 3.8 |
| RAA（cm²）,PSAX | 21 | 11 | 13 | 13 |
| RVD (cm), PSAX | 4.8 | 3.3 | 3.0 | 3.0 |
| TAPSE（cm） | 1.6 (z -3.85) | 1.9 (z -2.68) | 1.6 (z -4.15) | 1.6 (z -4.15) |
| PAAT（ms）,PSAX | 63 | 66 | 74 | 74 |
| LVEF (%) | 60 | 60 | 60 | 60 |
| RVMPI | 0.39 | 0.47 | 0.58 | 0.58 |
| Pericardial effusion | No | No | No | No |
| RV/LV | 1.38 | 0.82 | 1.06 | 1.06 |
| **Laboratory test** | | | | |
| NT-proBNP (pg/ml) | ＞30000.0 | 790.0 | 615.7 | 615.7 |
| ALT (U/L) | 44.0 | 9.0 | 9.0 | 9.0 |
| TBIL (μmol/L) | 12.3 | 18.6 | 14.4 | 14.4 |
| Cr (μmol/L) | 95.0 | 4.6 | 61.0 | 61.0 |
| UA (μmol/L) | 601.0 | 473.0 | 451.0 | 451.0 |
| HGB (g/L) | 124.0 | 135.0 | 129.0 | 129.0 |
| **Functional status** | | | | |
| WHO FC | Ⅲ | Ⅲ | Ⅱ | Ⅱ |
| 6MWD (m） | 240 | 316 | 435 | 435 |

Abbreviations: 6MWD, 6-minute walk distance; ALT, alanine aminotransferase; BMI, body mass index; BSA, body surface area; CI, cardiac index; Cr, creatinine; FC, functional class; HGB, haemoglobin; LVEF, left ventricular ejection fraction; MPI, myocardial performance index; NT-proBNP, N-terminal brain natriuretic peptide; PAH, pulmonary arterial hypertension; PAAT, pulmonary artery acceleration time; RAA, right atrial area; RAD, right atrial diameter; RV, right ventricle; RVD, right ventricular diameter; RV/LV, RV/LV end-systolic diameter; TAPSE, tricuspid annular plane systolic excursion; TBIL, total bilirubin; UA, uric acid; WHO, World Health Organization.

**#9**

|  | **Baseline** | **6-month** | **12-month** | **Last follow-up** |
| --- | --- | --- | --- | --- |
| **Demographics** | | | | |
| Age (years) | 16.9 | 17.4 |  | 17.4 |
| Height（cm） | 152.0 | 153.0 |  | 153.0 |
| Weight（kg） | 50.00 | 53.00 |  | 53.00 |
| BMI（kg/m²） | 21.64 | 22.64 |  | 22.64 |
| BSA（m²） | 1.41 | 1.46 |  | 1.46 |
| **Echocardiography (M-mode）** | | | | |
| RAD（cm）,PSAX | 7.5 | 6.4 |  | 6.4 |
| RAA（cm²）,PSAX | 48 | 35 |  | 35 |
| RVD (cm), PSAX | 4.8 | 5.0 |  | 5.0 |
| TAPSE（cm） | 1.0 (z -6.78) | 1.1 (z -6.59) |  | 1.1 (z -6.59) |
| PAAT（ms）,PSAX | 51 | 48 |  | 48 |
| LVEF (%) | 60 | 60 |  | 60 |
| RVMPI | 0.98 | 0.74 |  | 0.74 |
| Pericardial effusion | Yes | Yes |  | Yes |
| RV/LV | 2.61 | 2.35 |  | 2.35 |
| **Laboratory test** | | | | |
| NT-proBNP (pg/ml) | 2869.0 | 1291.0 |  | 1291.0 |
| ALT (U/L) | 60.0 | 9.0 |  | 9.0 |
| TBIL (μmol/L) | 39.6 | 28.2 |  | 28.2 |
| Cr (μmol/L) | 86.0 | 62.0 |  | 62.0 |
| UA (μmol/L) | 622.0 | 115.0 |  | 115.0 |
| HGB (g/L) | 88.0 | 110.0 |  | 110.0 |
| **Functional status** | | | | |
| WHO FC | Ⅳ | Ⅳ |  | Ⅳ |
| 6MWD (m） | 331 | 351 |  | 351 |

Abbreviations: 6MWD, 6-minute walk distance; ALT, alanine aminotransferase; BMI, body mass index; BSA, body surface area; CI, cardiac index; Cr, creatinine; FC, functional class; HGB, haemoglobin; LVEF, left ventricular ejection fraction; MPI, myocardial performance index; NT-proBNP, N-terminal brain natriuretic peptide; PAH, pulmonary arterial hypertension; PAAT, pulmonary artery acceleration time; RAA, right atrial area; RAD, right atrial diameter; RV, right ventricle; RVD, right ventricular diameter; RV/LV, RV/LV end-systolic diameter; TAPSE, tricuspid annular plane systolic excursion; TBIL, total bilirubin; UA, uric acid; WHO, World Health Organization.

**#10**

|  | **Baseline** | **6-month** | **12-month** | **Last follow-up** |
| --- | --- | --- | --- | --- |
| **Demographics** | | | | |
| Age (years) | 16.0 | 16.5 |  | 16.5 |
| Height（cm） | 172.0 | 175.0 |  | 175.0 |
| Weight（kg） | 68.00 | 70.00 |  | 70.00 |
| BMI（kg/m²） | 22.99 | 22.86 |  | 22.86 |
| BSA（m²） | 1.77 | 1.81 |  | 1.81 |
| **Echocardiography (M-mode）** | | | | |
| RAD（cm）,PSAX | 5.2 | 4.3 |  | 4.3 |
| RAA（cm²）,PSAX | 26 | 20 |  | 20 |
| RVD (cm), PSAX | 3.8 | 3.6 |  | 3.6 |
| TAPSE（cm） | 2.1 (z -1.41) | 2.2 (z -0.93) |  | 2.2 (z -0.93) |
| PAAT（ms）,PSAX | 94 | 88 |  | 88 |
| LVEF (%) | 60 | 60 |  | 60 |
| RVMPI | 0.45 | 0.41 |  | 0.41 |
| Pericardial effusion | Yes | Yes |  | Yes |
| RV/LV | 0.88 | 0.74 |  | 0.74 |
| **Laboratory test** | | | | |
| NT-proBNP (pg/ml) | 26.4 | 56.6 |  | 56.6 |
| ALT (U/L) | 10.0 | 14.0 |  | 14.0 |
| TBIL (μmol/L) | 8.4 | 3.0 |  | 3.0 |
| Cr (μmol/L) | 61.0 | 70.0 |  | 70.0 |
| UA (μmol/L) | 270.0 | 299.0 |  | 299.0 |
| HGB (g/L) | 145.0 | 156.0 |  | 156.0 |
| **Functional status** | | | | |
| WHO FC | Ⅱ | Ⅰ |  | Ⅰ |
| 6MWD (m） | 600 | 570 |  | 570 |

Abbreviations: 6MWD, 6-minute walk distance; ALT, alanine aminotransferase; BMI, body mass index; BSA, body surface area; CI, cardiac index; Cr, creatinine; FC, functional class; HGB, haemoglobin; LVEF, left ventricular ejection fraction; MPI, myocardial performance index; NT-proBNP, N-terminal brain natriuretic peptide; PAH, pulmonary arterial hypertension; PAAT, pulmonary artery acceleration time; RAA, right atrial area; RAD, right atrial diameter; RV, right ventricle; RVD, right ventricular diameter; RV/LV, RV/LV end-systolic diameter; TAPSE, tricuspid annular plane systolic excursion; TBIL, total bilirubin; UA, uric acid; WHO, World Health Organization.
